# P63-negative pulmonary NUT carcinoma arising in the elderly: a case report

**DOI:** 10.1186/s13000-020-01053-4

**Published:** 2020-11-11

**Authors:** Satoe Numakura, Koji Saito, Noriko Motoi, Taisuke Mori, Yuichi Saito, Fumi Yokote, Yasuyuki Kanamoto, Momoko Asami, Takashi Sakai, Yoshikane Yamauchi, Yukinori Sakao, Hiroshi Uozaki, Masafumi Kawamura

**Affiliations:** 1 Department of Pathology, Teikyo University Hospital, Tokyo, Japan; 2 Department of Pathology, Teikyo University School of Medicine, Tokyo, Japan; 3 Department of Diagnostic Pathology, National Cancer Center Hospital, Tokyo, Japan; 4 Department of Surgery, Teikyo University School of Medicine, Tokyo, Japan

**Keywords:** NUT carcinoma, Lung, NUT immunohistochemistry, p63, Case report

## Abstract

**Background:**

Pulmonary NUT carcinoma is rare, but lethal, thus, must not be overlooked. The definitive diagnosis is made by a NUT monoclonal antibody or gene analysis, but these are not always routinely available. Therefore, the diagnosis depends on this rare disease being suspected from the clinical and pathological findings. Generally, NUT carcinoma of the lung occurs near the hilum in younger adults with severe subjective symptoms. Histologically, it is characterized by the monomorphic growth of small cells which showed positivity of p63 immunohistochemistry.

**Case presentation:**

An 82-year-old man was referred for an incidental finding of an abnormal shadow at the peripheral apex of the right lung on computed tomography for a regular follow-up examination of renal cancer. Microscopically, small cell carcinoma was initially suspected; however, immunohistochemistry was not typical. NUT carcinoma with BRD4-NUT fusion was ultimately diagnosed using a NUT monoclonal antibody, fluorescence in situ hybridization, and RNA-seq. p63 and p40 protein expression was not detected.

**Conclusions:**

This is the first case of pulmonary NUT carcinoma to show negativity for p63 and is the oldest among previously reported cases. The present case suggests that NUT carcinoma should be suspected when the morphology of monomorphic growth of small cells without lineage-specific differentiation, regardless of age, clinical symptoms, the tumor location, or p63 expression.

**Supplementary Information:**

The online version contains supplementary material available at 10.1186/s13000-020-01053-4.

## Background

NUT midline carcinoma is rare, but lethal. It was initially considered to occur in the midline organs (the mediastinum and upper aerodigestive tract) of children or adolescents [1]. However, with the recognition of NUT midline carcinoma and emergence of novel NUT monoclonal antibodies, case numbers are now increasing in all ages and organs, including extra-midline sites (resulting in the name NUT carcinoma) [2]. The International NUT Midline carcinoma registry (www.nmcregistry.org) [3] was established in 2010 for the purpose of obtaining more information on this disease [4]. Therefore, there is an ever-increasing need to recognize this disease in all specialties.

NUT carcinoma is genetically defined and characterized by chromosomal rearrangements in the NUT gene. Its prognosis is poor regardless of the tumor site. There are currently no standard treatments and most cases are resistant to treatment; however, new therapeutic agents (histone deacetylase or BET inhibitors) are expected [1]. Historically, several epithelial malignancies with chromosomal translocation 15;19 were found to be clinically aggressive [5–7]. This chromosomal translocation has been shown to target the BRD4 gene [8] and NUT (Nuclear Protein in Testis) gene [9], which is the common fusion pair found in NUT midline carcinoma. More NUT fusion partners were subsequently identified (NUT variants) by the screening of carcinomas in younger patients [10]. This cancer showing NUT gene rearrangements has since been referred to as NUT midline carcinoma.

NUT midline carcinoma is diagnosed by immunohistochemistry with a NUT antibody (more than 50% positivity in tumor nuclei is considered to be diagnostic) or the demonstration of NUT rearrangements by fluorescence in situ hybridization (FISH), reverse-transcription PCR, cytogenetics, or next-generation sequencing [1]. Histologically, NUT midline carcinoma is characterized by the monomorphic growth of small round- to oval-shaped cells. Nuclear mitoses and necrosis are frequently reported [11]. Squamous differentiation may be present in some cases [11], but not in others [2]. Although NUT carcinoma shows no specific findings on general immunostaining, carcinoma cells express the p63 protein in most cases. Thus, difficulties are associated with reaching a diagnosis without recognizing the existence of this rare disease, particularly in institutions in which NUT immunohistochemistry and gene analyses are not available.

NUT midline carcinoma may occur in the lung. NUT midline carcinoma of pulmonary origin is classified as NUT carcinoma in the 2015 World Health Organization (WHO) classification of lung tumors [12]. We herein report a case of pulmonary NUT carcinoma in an elderly patient.

## Case presentation

An 82-year-old man was referred for an abnormal shadow detected 4 months previously on computed tomography (CT) in a regular follow-up after bilateral clear cell renal cell carcinoma. He underwent left nephrectomy 14 years ago and right partial nephrectomy 5 years ago. At that time, he stated that he had a prolonged cough for several months. He had a smoking history of a half pack-year for 20 years. His father had gastric cancer, his mother had thyroid cancer, and his son had a brain tumor. He was a merchant and had no exposure history to asbestos. He had a bird mamelliha.

**Fig. 1:**
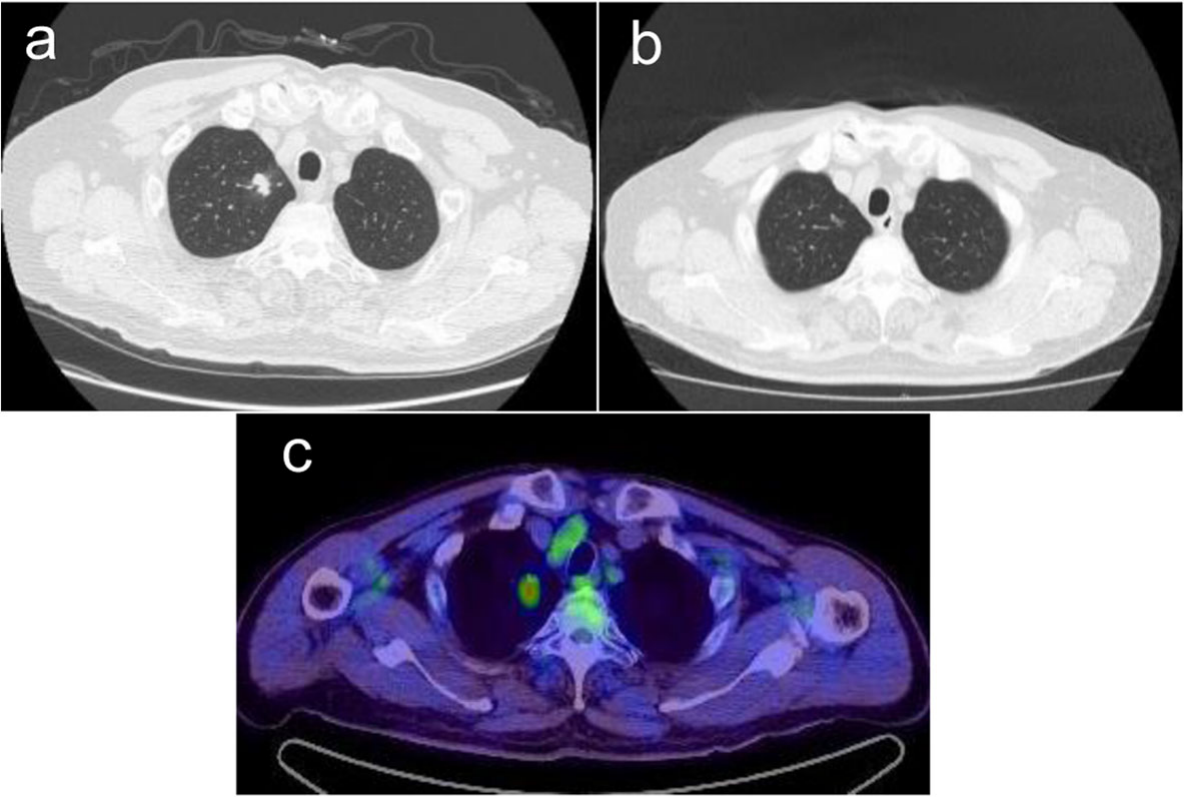
CT and PET/CT scans revealed a mass in the apex of the right lung. Chest CT showed a mass in the upper lobe of the right lung (**a**), which was not detected 2 months previously (**b**). In the PET/CT scan, this mass showed abnormal accumulation (**c**)

Chest CT showed a 14-mm nodular shadow on the apex of the right upper lobe of the lung with no lymphadenopathy or pleural effusion. PET/CT (positron emission tomography-computed tomography) revealed abnormal accumulation in 2 nodules of the upper lobe of the right lung and ipsilateral hilar- and mediastinal lymph nodes (Fig. 1). Abdominal CT and brain MRI (magnetic resonance imaging) were negative for metastasis. Laboratory examinations showed elevated levels of serum progastrin-releasing peptide [ProGRP; 115 pg/mL (normal range: 0–70.9 pg/mL)] and soluble IL-2 receptor [S.IL-2R; 992 U/mL (normal range: 122–496 U/mL)], and a slightly elevated level of squamous cell carcinoma antigen [SCC; 2.7 ng/mL (normal range: 0–2.5 ng/mL)]. Serum levels of carcinoembryonic antigen (CEA) and cytokeratin-19 fragments (CYFRA) were within normal limits. Lung metastasis from renal cancer or primary lung cancer was clinically suspected and the patient underwent partial resection of the upper lobe of the right lung via video-assisted thoracotomy.

**Fig. 2:**
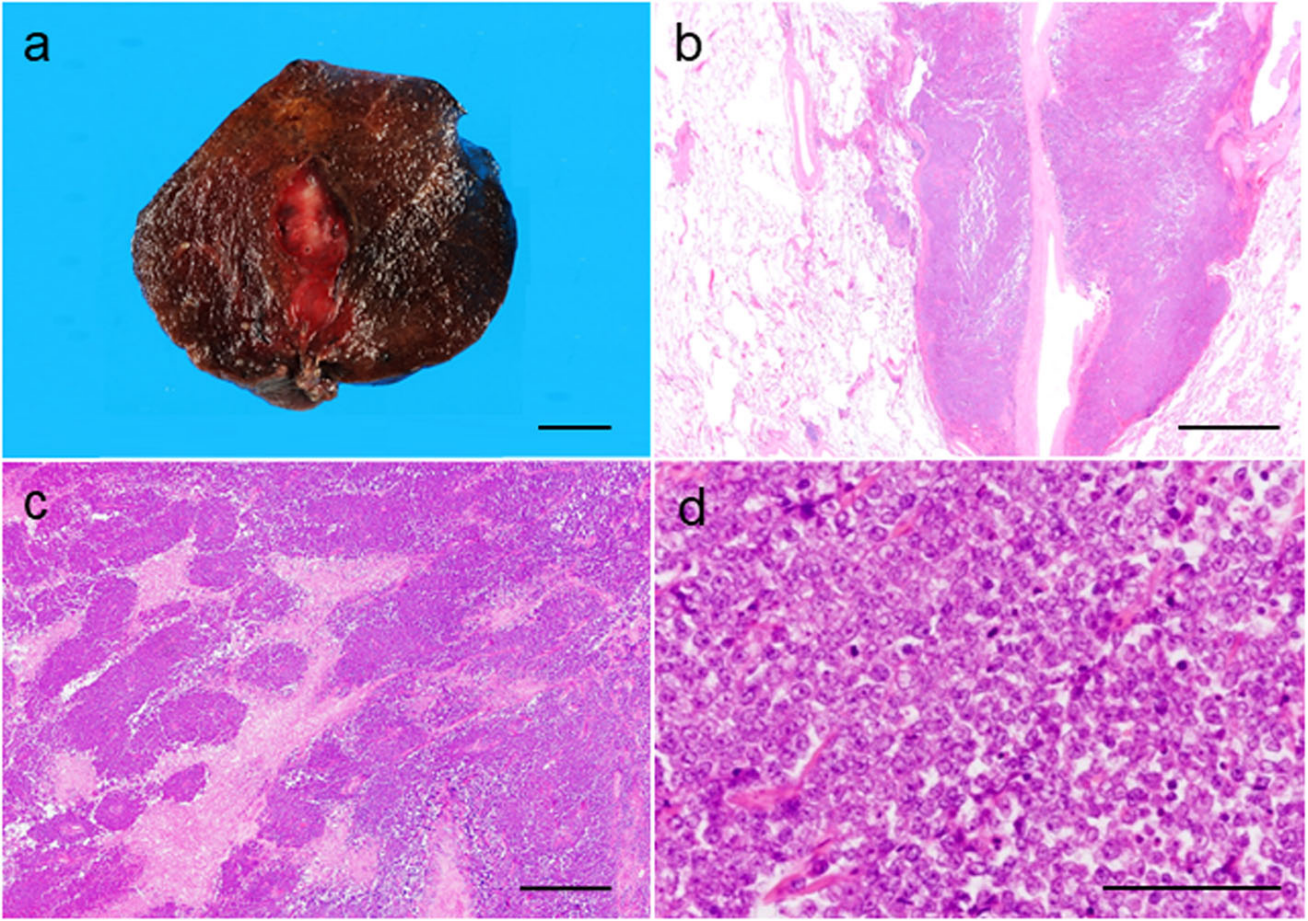
Gross and histological findings of the pulmonary tumor. **a** Gross cut section. A white solid tumor with an irregular border and 25 × 12 × 10 mm in size. Bar, 10 mm. **b** Low-power view showing the tumor mainly in the soft tissue of the bronchial vascular bundle. The structure of the pulmonary artery was maintained inside the tumor. Hematoxylin and eosin (HE) stain. Bar, 2 mm. **c** Necrosis within the tumor. HE stain. Bar, 500 μm. **d** High-power view showing the sheet growth of monomorphic small round cells. Tumor cells had round to oval nuclei with distinct nucleoli and a scanty cytoplasm. Mitotic figures were easily found. HE stain. Bar, 100 μm

**Fig. 3:**
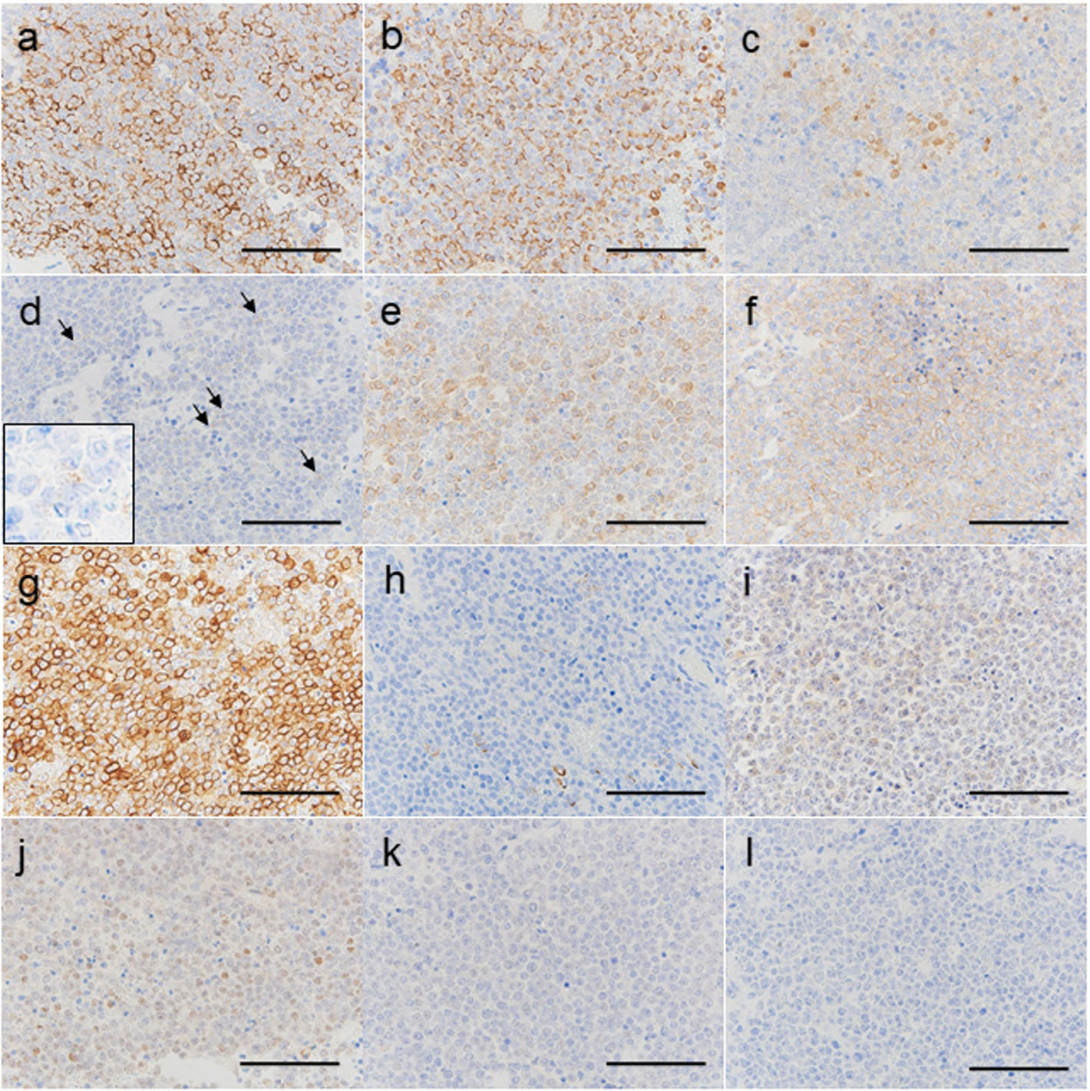
Representative immunohistochemical findings of the pulmonary tumor. Tumor cells were positive to various degrees for EMA (**a**), Vimentin (**b**), NSE (**c**), Chromogranin A (**d** arrows and inset), BCL-2 (**e**), CD99 (**f**), HHF35 (**g**), Desmin (**h**), Calponin (**i**) and SALL4 (**j**). P63 (**k**) and p40 (**l**) were negative. Bar, 100 μm

A gross examination revealed a solid tumor with an irregular border, measuring 25 × 12 × 10 mm with a white cut surface (Fig. 2). Paraffin sections showed that the tumor was composed of sheets of monomorphic small round cells with necrosis, which was not consistent with typical clear cell renal cell carcinoma (Fig. 2). There was no obvious or abrupt squamous differentiation or tube formation. Tumor cells had round- to oval-shaped nuclei with distinct nucleoli and a scanty cytoplasm. Mitotic figures (27/20 HPF) were clearly observed. There was lymphatic invasion and two intrapulmonary metastases of 2 and 1 mm in size. These intrapulmonary metastases were detected in the sub-pleura 8 mm from the main tumor. Primary and intrapulmonary metastatic lesions both extended into the soft tissue of bronchial vascular bundles and the structure of the pulmonary artery was maintained inside the tumor (Fig. 2). There was no pleural invasion, pleural dissemination, or venous infiltration. The surgical margin was negative. We initially suspected small cell carcinoma; however, immunohistochemistry was less typical (Fig. 3) because tumor cells were focally positive for neuron-specific enolase (NSE) and chromogranin A and negative for CD56 and synaptophysin. Although we searched for lymphoma, tumor cells were only focally positive for B-cell lymphoma-2 (BCL-2) and negative for other lymphocytic markers (leukocytic common antigen (LCA), CD3, CD5, terminal deoxynucleotidyl transferase (TdT), CD10, CD20, CD79a, and cyclin D1). Due to the focal positivity of BCL-2, we suspected synovial sarcoma as a differential diagnosis and attempted to exclude other diseases, but were unable to reach a final diagnosis based on general immunohistochemistry alone. Tumor cells were positive for epithelial membrane antigen (EMA) and vimentin and focally positive for CD99, HHF-35, desmin, calponin, and Sal-like protein 4 (SALL4). They were negative for AE1/AE3, cytokeratin 7 (CK7), cytokeratin 20 (CK20), cytokeratin 5/6 (CK5/6), CAM5.2, thyroid transcription factor 1 (TTF-1), tumor protein p63 (p63), p40, myoblast determination protein 1 (MyoD1), caldesmon, glypican 3 (GPC3), placental alkaline phosphatase (PLAP), calretinin, S-100, CD117 [c-kit], Melan A, HMB-45, Wilm’s tumor suppressor gene 1 (WT-1), paired box gene 8 (PAX8), and D2–40. EBER in situ hybridization was also negative. Our differential diagnoses for these small round cell tumors were small cell carcinoma, germ cell tumor, soft tissue sarcoma (synovial sarcoma, extra-skeletal Ewing’s sarcoma, Ewing-like sarcoma [CIC rearrangement sarcoma, and sarcoma with BCOR genetic alterations], and malignant glomus tumor), and NUT carcinoma.

**Fig. 4:**
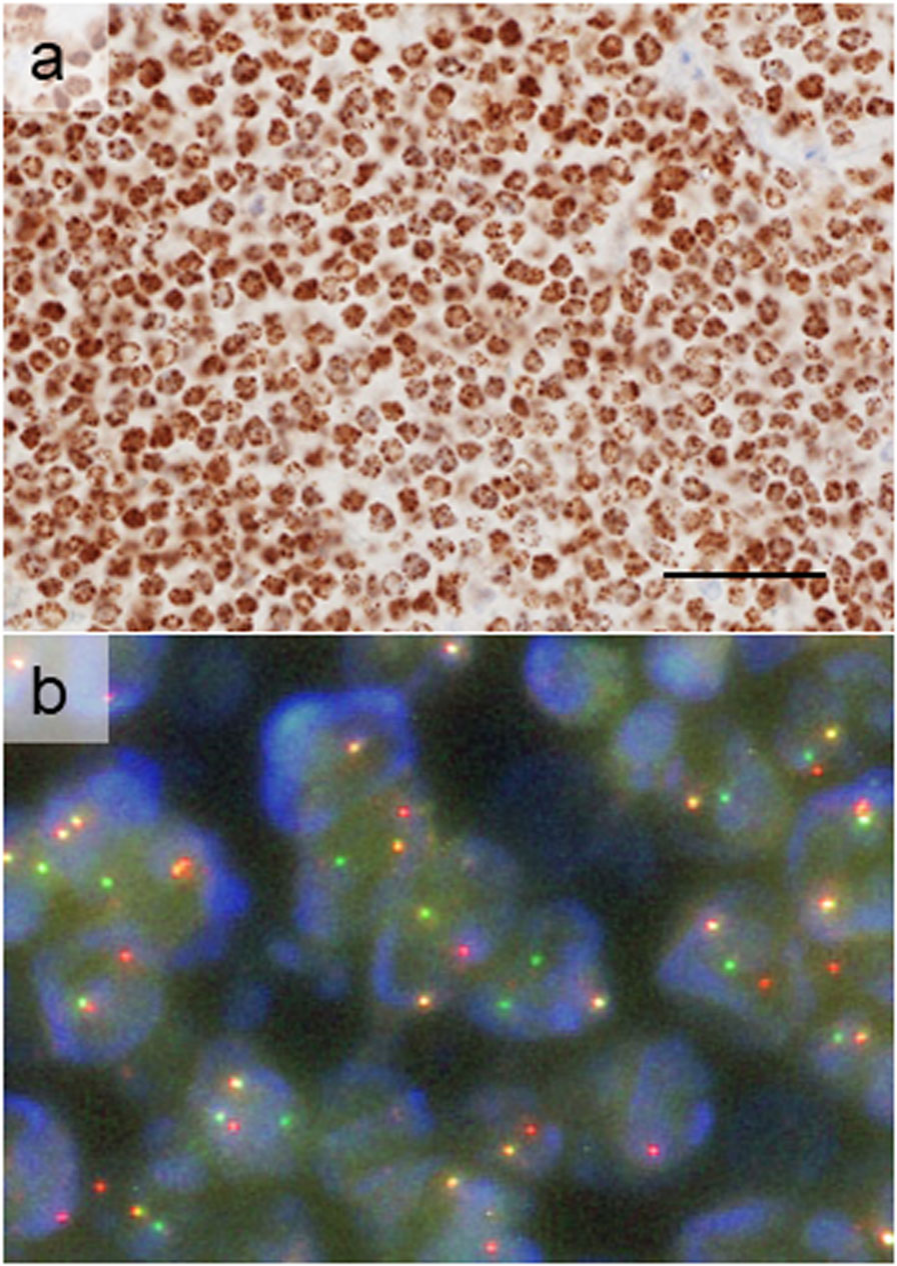
NUT immunohistochemistry and Fluorescence in situ hybridization. **a** NUT immunohistochemistry. Diffusely positive tumor nuclei with a speckled pattern. Bar, 100 μm. **b** Fluorescence in situ hybridization (FISH). The BRD4-NUT fusion gene was detected

We subsequently performed NUT immunohistochemistry (rabbit monoclonal, C52B1, × 100, TRSpH9, Cell Signaling) using AutostainerLink48, which showed diffusely positive tumor nuclei with a speckled pattern. FISH and RNA sequencing (RNA-Seq) revealed the presence of the BRD4-NUT fusion gene (Fig. 4). These results led to the final diagnosis of primary pulmonary NUT midline carcinoma with the BRD4-NUT fusion (pT3NxM0).

## Discussion and conclusions

Pulmonary NUT carcinoma is rare. A search using PubMed and Google scholar with the key words NUT (midline) carcinoma, pulmonary or lung (Additional file 1) showed that 55 case reports have been published in English to date. Its rarity is attributed to its occurrence and lack of recognition [13]. This rare disease is diagnosed by a NUT monoclonal antibody or gene analysis. However, since not all institutions always have the necessary equipment to perform these analyses, requests are made to other facilities. Under these conditions, the diagnosis of NUT carcinoma depends on this disease being suspected at the clinical and morphological stages. Primary pulmonary NUT carcinoma has a very poor prognosis, with a median survival time of 2.2 months (66 days) [14].

NUT carcinoma may be suspected based on clinical characteristics [15]. However, the present case was not typical of the clinical features of pulmonary NUT carcinoma in age, size, or location. Pulmonary NUT carcinoma cases have frequently been reported in younger patients with a median age of 30 years [14]. Our patient was the oldest reported case of NUT carcinoma of pulmonary origin and also of various origins [11, 16]. The majority of previously reported cases of pulmonary NUT carcinoma were larger in size, centrally located, and identified based on strong subjective symptoms. However, the size of the tumor in the present case was smaller and peripherally located. Although an interview with our patient revealed a prolonged cough, the initial opportunity to detect the tumor was in a follow-up examination for renal cancer, and not based on subjective symptoms.

On the other hand, the morphology of the tumor in the present case was consistent with previously reported characteristics, but less specific. Since there was a small monotonous morphology without specific differentiation, we initially suspected small cell carcinoma; however, neuroendocrine marker expression was not typical. NUT midline carcinoma sometimes expresses neuroendocrine markers to various degrees. Previous studies reported that it was hidden in small cell lung cancer [15, 17]. Based on these findings and previous cases with information on neuroendocrine marker expression, including the present case (Additional file 1), we speculated whether pulmonary NUT carcinoma needs to be considered when less than one out of 3 neuroendocrine markers (chromogranin A, synaptophysin, and CD56) are (incompletely) positive in tumors showing a monotonous morphology. In previously reported cases of pulmonary NUT carcinoma with an examination of p63 expression, all cases showed positivity for p63 to various degrees (Additional file 1). The present case is the first case report of pulmonary NUT carcinoma showing negativity for p63 expression.

It is important to note that the tumor in the present case appeared to only invade bronchial bundle tissue, which has not been previously reported. A previous study hypothesized that NUT midline carcinoma arises from early epithelial progenitor-cell rests [10]. The present case was positive for EMA and negative for other cytokeratin markers. These results suggest that NUT carcinoma cells in the present case originated from epithelial-like immature cells in the soft tissue of the lung.

In conclusion, the present case indicates the importance of recognizing NUT carcinoma, and in the case of tumors showing monomorphism composed of immature small cells without an obvious specific lineage differentiation, NUT immunohistochemistry needs to be considered regardless of age, clinical symptoms, the tumor location, and p63 expression.

## Supplementary Information

**Additional file 1.** Published primary pulmonary NUT carcinoma cases and the present case. Abbreviations: N/A indicates not available.

## Data Availability

Not applicable.

## Abbreviations

NUT: Nuclear protein in testis
FISH: Fluorescence in situ hybridization
CT: Computed tomography
PET/CT: Positron emission tomography-computed tomography
MRI: Magnetic resonance imaging
ProGRP: Progastrin-releasing peptide
SCC: Squamous cell carcinoma antigen
CEA: Carcinoembryonic antigen
CYFRA: Cytokeratin-19 fragments
NSE: Neuron-specific enolase
BCL-2: B-cell lymphoma-2
LCA: Leukocytic common antigen
CD3: Cluster of differentiation 3
CD5: Cluster of differentiation 5
CD10: Cluster of differentiation 10
CD20: Cluster of differentiation 20
CD79a: Cluster of differentiation 79a
CD56: Cluster of differentiation 56
CD99: Cluster of differentiation 99 (CD99)
CD117: Cluster of differentiation 117
CK7: Cytokeratin 7
CK20: Cytokeratin 20
CK5/6: Cytokeratin 5/6
TdT: Terminal deoxynucleotidyl transferase
EMA: Epithelial membrane antigen
SALL4: Sal-like protein 4
TTF-1: Thyroid transcription factor 1
MyoD1: Myoblast determination protein 1
GPC3: Glypican 3
PLAP: Placental alkaline phosphatase
WT-1: Wilm’s tumor suppressor gene 1
PAX8: Paired box gene 8
p63: Tumor protein p63
